# Association between digital dermatoglyphics and handedness among Sinhalese in Sri Lanka

**DOI:** 10.12688/f1000research.2-111.v3

**Published:** 2013-11-04

**Authors:** Buddhika TB Wijerathne, Geetha K Rathnayake

**Affiliations:** 1 Department of Forensic Medicine, Faculty of Medicine and Allied Sciences, Rajarata University of Sri Lanka, Saliyapura, Sri Lanka; 2 Teaching Hospital Anuradhapura, Anuradhapura, 50000, Sri Lanka

**Keywords:** Forensic science, handedness, digital dermatoglyphics, fingerprints, personal identification, Sinhalese, Sri Lankans

## Abstract

**Background ** The relationship between handedness and digital dermatoglyphic patterns has never been investigated in the Sinhalese population. The goal of this study is to establish the above mentioned relationship, which would positively aid personal identification.

**Findings** One hundred forty Sinhalese students (70 right-handed and 70 left-handed) were studied for their digital dermatoglyphic pattern distribution. The results show that a statistically significant correlation exists for; digit 5 (Ulnar loop; P= 0.0449 and radial loop; P= 0.0248 by Fisher’s exact test) of the right hand in female, digit 1 (radial loop; P=0.0248 by Fisher’s exact test) and digit 2 (Ulnar loop; P=0.0306) of the left hand in females, digit 3 (Ulnar loop; P= 0.0486 and whorl; P= 0.0356 by Fisher’s exact test) and digit 4 (Ulnar loop; P= 0.0449 and whorl; P= 0.0301 by Fisher’s exact test) of the right hand in males, digit 4 (whorl; P=0.0160 by Fisher’s exact test) of the left hand in males.

**Conclusions ** Statistically significant differences in handedness and digital dermatoglyphic patterns were evident among Sinhalese people. Further study with a larger sample size is recommended.

## Introduction

Fingerprints (digital dermatoglyphics) are a unique form of evidence that greatly contribute towards personal identification in forensic science ^1^. Because they are unique for each individual and are strongly influenced by genetics, they also perform a significant role in anthropology, human genetics, ethnology and medicine. They are characterized by alternating strips of raised friction ridges and grooves present in a variety of patterns ^2^. These patterns start to develop between the 5th and 6th week of intrauterine life, and are fully formed by the 21st week ^3^. These patterns do not change throughout postnatal life and their development is determined by several genes ^4^.

Handedness (i.e. hand dominance) is defined as the uneven distribution of fine motor skills between the left and right hand ^5^. Determination of the handedness of both the assailant and the victim are important in various aspects of forensic science, including personal identification ^6^. Hence, establishing the relationship between handedness and digital dermatoglyphics will aid forensic identification.

To date, scarce amount of studies ^7– 13^ have investigated whether there is a correlation between handedness and digital dermatoglyphics. In 1940 Cummins discovered a slight association in the sex differences of asymmetrical occurrence of dermatoglyphic patterns ^8^. Cromwell and Rife in 1942 found that left-handers are characterized by slightly less bimanual asymmetry than right-handers among on Caucasian school children in southwestern Ohio ^9^. In 1943 Rife found associations characteristic of autosomal linkage between the whorl frequencies on the fingers and handedness among descended from northern European stock ^10^. In 1994 Coren reported an increased number of arches, fewer whorls in left-handers as compared to the right-handers among Canadians ^11^. Cho in 2010 found significant difference of dermatoglyphics patterns on digit 3, 4 and 5 among Koreans ^12^. None have investigated this association in a Sinhalese population (an Indo-Aryan ethnic group who are native to the island of Sri Lanka ^14^). The main goal of the current study is to determine the relationship between handedness and digital dermatoglyphics in a sample of Sinhalese population.

## Methods

The study was conducted at the Department of Forensic Medicine, Faculty of Medicine and Allied Sciences, Rajarata University of Sri Lanka. Ethical clearance for this study was obtained from the Ethical Clearance Committee of the institute. Total of hundred forty Sinhalese students (70 females, 70 males) who gave informed written consent were included in the study. Ages of females ranged between 21 and 28 years (mean ± s.d. = 24.40 ± 1.82 years) and males ranged from 22 and 28 years (mean ± s.d. = 24.67 ± 1.92 years). Firstly, handedness was assessed using the Edinburgh Handedness Inventory ^15^. This required participants to demonstrate 10 unimanual tasks (preferred hand for writing, drawing, throwing, striking a match, opening a box, holding scissors, holding a toothbrush, holding a spoon, holding a broom and holding a knife). These tasks are common to Sri Lankans and they were advised to state the degree of preference for the hand used in each case as either strong (two points) or weak (one point). The handedness measure was calculated by subtracting the score for the left hand from the score for the right hand, dividing by the sum of both, and multiplying it by 100, providing an absolute range from -100 (completely left-handed) to +100 (completely right-handed). We recruited 70 predominant right-handers and 70 predominant left-handers after evaluating handedness.

**Figure 1.**
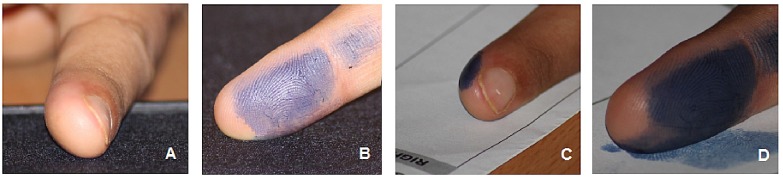
Method for obtaining fingerprints. **A** and **B** show the rolling of the finger from the radial side to ulnar side on an ink pad. **C** and **D** show the transference of fingerprints onto the allocated area of the paper.

All eligible students were asked to wash their hands thoroughly to remove dirt and dry them before obtaining fingerprints. Rolled prints were obtained by the ink and paper method as described by Cummins and Midlo ^2^. The subject was asked to roll their finger from the radial side to the ulnar side on an ink pad and then transfer their fingerprints in the same manner onto the allocated area of a double sheet of plain A4 paper ( Figure 1). In this way, fingerprints for all the ten fingers were obtained for each individual. Digits are numbered as follows; digit 1 (thumb), digit 2 (index finger), digit 3 (middle finger), digit 4 (ring finger) and digit 5 (little finger).

**Figure 2.**
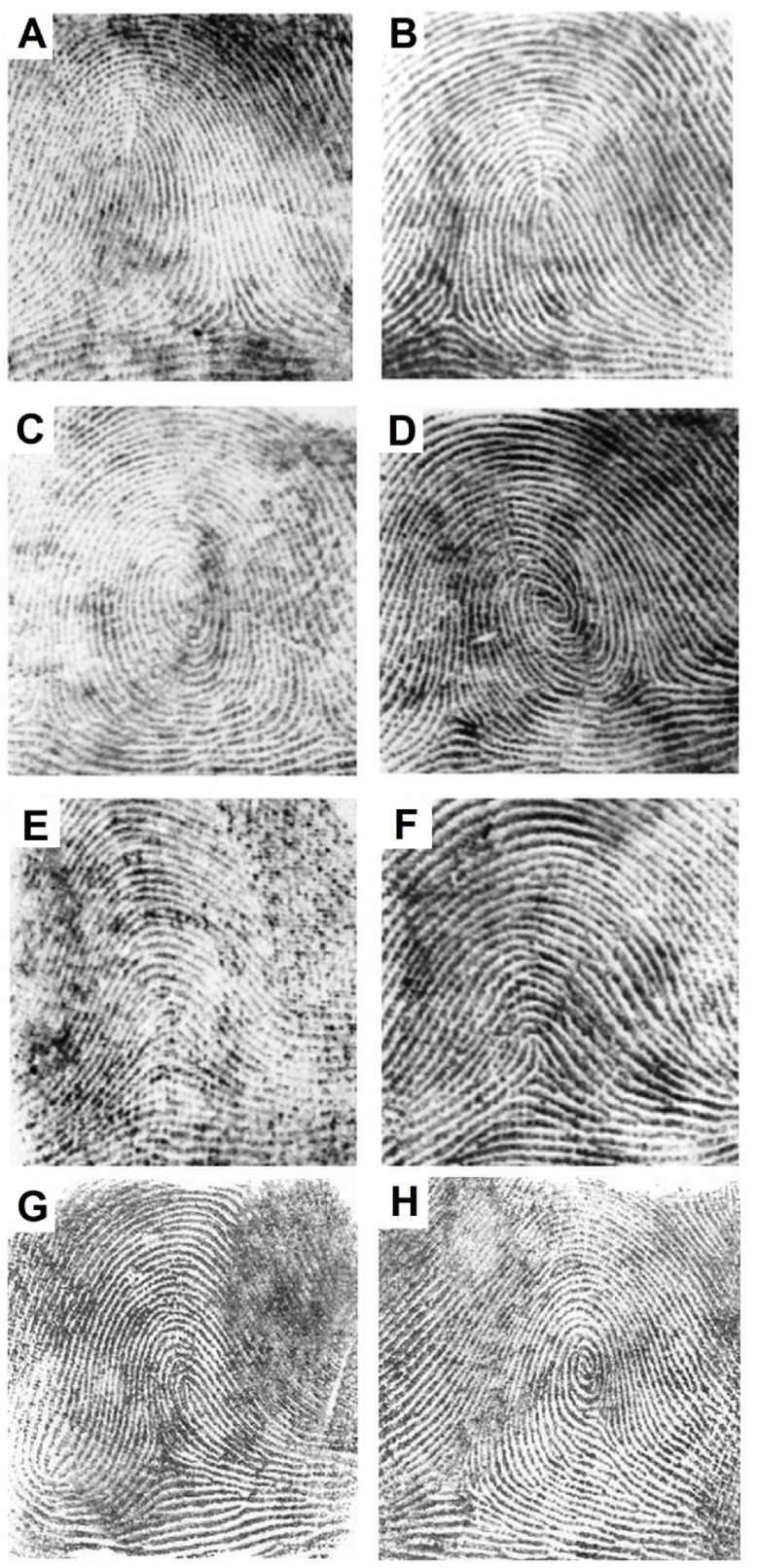
Different types of fingerprints. **A**: Ulnar loop, **B**: Radial loop, **C**: Plain Whorl, **D**: Double loop whorl, **E**: Plain arch, **F**: Tented arch, **G**: Accidental whorl, **H**: Central pocket loop.

Digital dermatoglyphic patterns ( Figure 2) were classified as follows; ulnar loop, radial loop, whorl (double loop whorl, plain whorl, central pocket loop and accidental whorl were counted as whorl) and arch (plain arch and tented arch were counted as arch ). In this way, fingerprints of all the ten fingers were obtained for each individual.

Analysis was carried out using GraphPad Prism 5 software (version 5.03 for Windows; GraphPad Software, San Diego California USA). Descriptive statistics were used to express the data. Correlations between handedness and digital dermatoglyphics were evaluated by a two-sided Fisher’s exact test. P values less than 0.05 were considered statistically significant.

## Results

In this study we observed the handedness-wise digital dermatoglyphics pattern distribution of 140 individuals (70 left-handed [35 females, 35 males] and 70 right-handed [35 males, 35 females]).

### Handedness wise differences of digital prints in females

***Right hand.*** Table 1 shows the digital dermatoglyphic pattern distribution of the right hand in females. On the digit 3 of right hand of right-handed students found to have more ulnar loop (74%) compared to left handers (57%) and on the digit 5 of right hand of right-handed students found to have more ulnar loop (77%) compared to left handers (51%). On the digit 5 of right hand of left-handed students found to have more radial loop (17%) compared to right handers (0%). Whorl and arch patterns have not shown significant difference. A statistically significant correlation was observed in digital dermatoglyphic patterns between right and left-handed people for digit 5 (Ulnar loop; P = 0.0449 and radial loop; P = 0.0248 by Fisher’s exact test).

**Table 1.** Digital dermatoglyphic pattern distribution of right hand in females.

Digit	Handedness	Ulnar Loop					Radial Loop					Whorl					Arch				
		(+)		(-)		P value‡	(+)		(-)		P value‡	(+)		(-)		P value‡	(+)		(-)		P value‡
		n	%	n	%		n	%	n	%		n	%	n	%		n	%	n	%	
Digit 1	Right	23	66	12	34	0.6238	0	0	35	100	0.2391	12	34	23	66	1	0	0	35	100	1
	Left	20	57	15	43		3	9	32	91		11	31	24	69		1	3	34	97	
Digit 2	Right	21	60	14	40	1	0	0	35	100	0.4928	11	31	24	69	1	3	9	32	91	0.6139
	Left	22	63	13	37		2	6	33	94		10	29	25	71		1	3	34	97	
Digit 3	Right	26	74	9	26	0.2076	2	6	33	94	0.4283	7	20	29	83	0.5798	0	0	35	100	1
	Left	20	57	15	43		5	14	30	86		9	26	26	74		1	3	34	97	
Digit 4	Right	19	54	16	46	1	1	3	34	97	1	15	43	20	57	0.6238	0	0	35	100	0.4928
	Left	19	54	16	46		2	6	33	94		12	34	23	66		2	6	33	94	
Digit 5	Right	27	77	8	23	0.0449*	0	0	35	100	0.0248*	8	23	27	77	0.7851	0	0	35	100	1
	Left	18	51	17	49		6	17	29	83		10	29	25	71		1	3	34	97	

‡ = Two sided fishers exact test, * P=<0.05

***Left hand.*** Table 2 shows the digital dermatoglyphic pattern distribution of the left hand in females. On the digit 3 of left hand of right-handed students found to have more ulnar loop (71%) compared to left handers (54%) and on the digit 5 of left hand of right-handed students found to have more ulnar loop (69%) compared to left handers (49%). On the digit 2 of left hand of left-handed students found to have more ulnar loop (63%) compared to left handers (34%), followed by 40% whorl on right handed compared to 23% whorl in left handed. On the digit 1 of left hand of right-handed individuals found to have more whorl (46%) compared to left handers (29%), followed by 17% radial loop on left handed compared to 0 % radial loop in right handed. A statistically significant correlation was observed in digital dermatoglyphic patterns between right and left-handed people for digit 1 (Radial loop; P = 0.0248 by Fisher’s exact test) and digit 2 (Ulnar loop; P = 0.0306 by Fisher’s exact test).

**Table 2.** Digital dermatoglyphic pattern distribution of left hand in females.

Digit	Handedness	Ulnar Loop					Radial Loop					Whorl					Arch				
		(+)		(-)		P value‡	(+)		(-)		P value‡	(+)		(-)		P value‡	(+)		(-)		P value‡
		n	%	n	%		n	%	n	%		n	%	n	%		n	%	n	%	
Digit 1	Right	17	49	18	51	0.8112	0	0	35	100	0.0248*	16	46	19	54	0.2159	2	6	33	94	0.4928
	Left	19	54	16	46		6	17	29	83		10	29	25	71		0	0	35	100	
Digit 2	Right	12	34	23	66	0.0306*	5	14	30	86	0.7096	14	40	21	60	0.1975	4	11	31	89	0.6733
	Left	22	63	13	37		3	9	32	91		8	23	27	77		2	6	33	94	
Digit 3	Right	25	71	10	29	0.2159	1	3	34	97	0.3565	7	20	28	80	0.5781	2	6	33	94	1
	Left	19	54	16	46		4	11	31	89		10	29	25	71		2	6	33	94	
Digit 4	Right	18	51	17	49	1	1	3	34	97	0.3565	16	46	19	54	0.3261	0	0	35	100	1
	Left	19	54	16	46		4	11	31	89		11	31	24	69		1	3	34	97	
Digit 5	Right	24	69	11	31	0.1449	1	3	34	97	0.3565	9	26	26	74	0.4403	1	3	34	97	1
	Left	17	49	18	51		4	11	31	89		13	37	22	63		1	3	34	97	

‡ = Two sided fishers exact test, * P = <0.05

### Handedness wise differences of digital prints in males

***Right hand.*** Table 3 shows the digital dermatoglyphic pattern distribution of the right hand in males. On the digit 3 of right hand of right-handed students found to have more ulnar loop (74%) compared to left handers (49%) and on the digit 4 of right hand of right-handed students found to have more ulnar loop (49%) compared to left handers (23%). On the digit 3 of right hand of left-handed students found to have more whorl (43%) compared to right-handers (17%) and on the digit 4 of right hand of left-handed students found to have more whorl (69%) compared to right handers (40%). Radial loop and arch pattern have not shown significant difference. A statistically significant correlation was observed in digital dermatoglyphic patterns between right and left-handed people for digit 3 (Ulnar loop; P = 0.0486 and whorl; P = 0.0356 by Fisher’s exact test) and digit 4 (Ulnar loop; P = 0.0449 and whorl; 0.0301 by Fisher’s exact test).

**Table 3.** Digital dermatoglyphic pattern distribution in right hand of males.

Digit	Handedness	Ulnar Loop					Radial Loop					Whorl					Arch				
		(+)		(-)		P value‡	(+)		(-)		P value‡	(+)		(-)		P value‡	(+)		(-)		P value‡
		n	%	n	%		n	%	n	%		n	%	n	%		n	%	n	%	
Digit 1	Right	20	57	15	43	1	2	6	33	94	0.4928	13	37	22	63	1	0	0	35	100	0.4928
	Left	19	54	16	46		0	0	35	100		14	40	21	60		2	6	33	94	
Digit 2	Right	19	54	16	46	1	3	9	32	91	1	9	26	26	74	1	4	11	31	89	1
	Left	18	51	17	49		4	11	31	89		9	26	26	74		4	11	31	89	
Digit 3	Right	26	74	9	26	0.0486*	1	3	34	97	1	6	17	29	83	0.0356*	2	6	33	94	1
	Left	17	49	18	51		2	6	33	94		15	43	20	57		1	3	34	97	
Digit 4	Right	17	49	18	51	0.0449*	4	11	31	89	0.6733	14	40	21	60	0.0301*	0	0	35	100	1
	Left	8	23	27	77		2	6	33	94		24	69	11	31		1	3	34	97	
Digit 5	Right	26	74	9	26	1	4	11	31	89	0.1142	5	14	30	86	0.3707	0	0	35	100	1
	Left	26	74	9	26		0	0	35	100		9	26	26	74		0	0	35	100	

‡ = Two sided fishers exact test, * P = <0.05

***Left hand.*** Table 4 shows the digital dermatoglyphic pattern distribution of the right hand in males. On the digit 2 of left hand of left-handed students found to have more ulnar loop (63%) compared to right handers (43%) and on the digit 4 of left hand of right-handed students found to have more ulnar loop (60%) compared to left handers (37%). Radial loop, whorl and arch pattern have not shown significant difference. A statistically significant correlation was observed in digital dermatoglyphic patterns between right and left-handed people for digit 4 (0.016 by Fisher’s exact test).

**Table 4.** Digital dermatoglyphic pattern distribution of left hand in males.

Digit	Handedness	Ulnar Loop					Radial Loop					Whorl					Arch				
		(+)		(-)		P value‡	(+)		(-)		P value‡	(+)		(-)		P value‡	(+)		(-)		P value‡
		n	%	n	%		n	%	n	%		n	%	n	%		n	%	n	%	
Digit 1	Right	20	57	15	43	0.3185	2	6	33	94	1	13	37	22	63	0.2968	0	0	35	100	1
	Left	25	71	10	29		1	3	34	97		8	23	27	77		1	3	34	97	
Digit 2	Right	15	43	20	57	0.1503	4	11	31	89	0.6733	11	31	24	69	0.1535	5	14	30	86	1
	Left	22	63	13	37		2	6	33	94		5	14	30	86		6	17	29	83	
Digit 3	Right	21	60	14	40	0.6307	3	9	32	91	1	8	23	27	77	0.4279	3	9	32	91	1
	Left	18	51	17	49		2	6	33	94		12	34	23	66		3	9	32	91	
Digit 4	Right	21	60	14	40	0.0935	3	9	32	91	0.2391	11	31	24	69	0.016*	0	0	35	100	1
	Left	13	37	22	63		0	0	35	100		22	63	13	37		0	0	35	100	
Digit 5	Right	25	71	10	29	0.5781	3	9	32	91	0.2391	7	20	28	80	1	0	0	35	100	1
	Left	28	80	7	20		0	0	35	100		7	20	28	80		0	0	35	100	

‡ = Two sided fishers exact test, * P = <0.05

**Figure 3.**
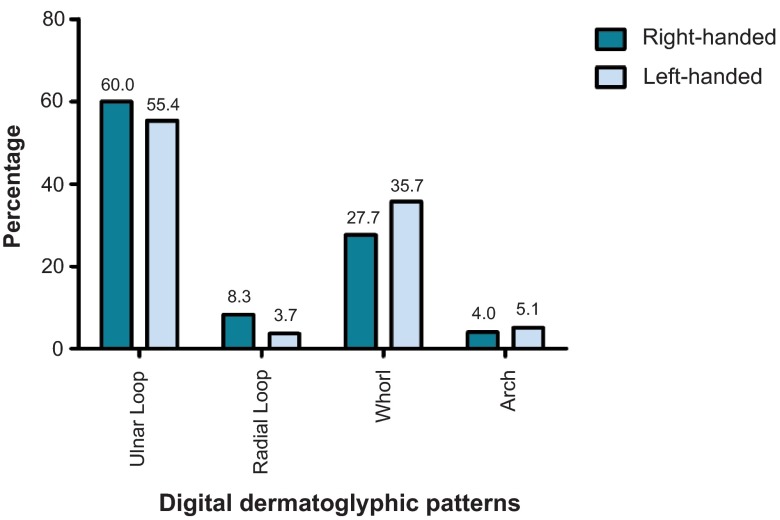
Digital dermatoglyphics pattern distributions in both hands of males.

**Figure 4.**
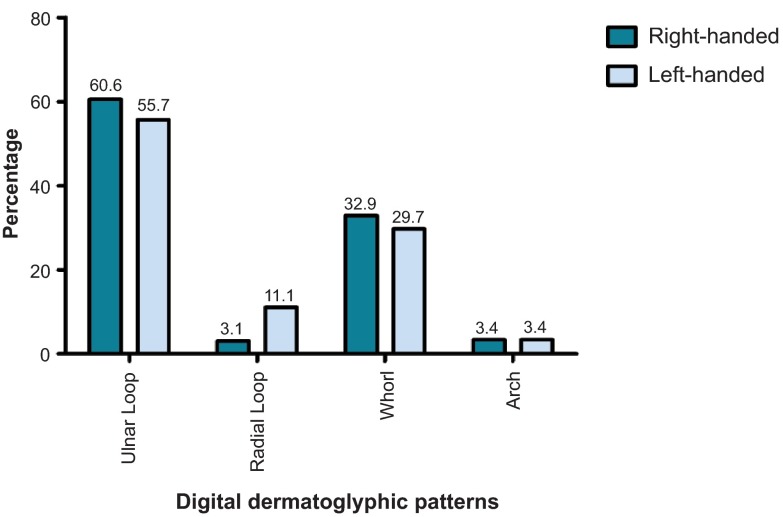
Digital dermatoglyphics pattern distributions in both hands of females.

The percentage of digital dermatoglyphics pattern distributions for both hands in male and female Sinhalese are shown in Figure 3 and Figure 4.

## Discussion

It has been affirmed that the digital dermatoglyphic pattern of the skin is unique and unchallengeable for an individual ^1^. This is valuable as a means of identification. In this study, effort has been made to study the relationship between dermatoglyphic and handedness in 140 Sinhalese students.

The results showed that a statistically significant correlation exists in digit 5 of the right hand while digit 1 and digit 2 of left hand in female. In males digit 3 and digit 4 of right hand and digit 4 of left hand showed a statistically significant correlation.

In the past, few studies have been conducted on different ethnic groups with the idea of establishing a relationship between handedness and dermatoglyphic pattern. Results of some studies are in line with the present study.

In their study on Caucasian school children in southwestern Ohio, Cromwell and Rife (1942) ^9^ observed a slightly higher frequency of whorls (1.3%) on left ring fingers (digit 4) of left-handers than of right-handers. Whorls were absent on the right ring finger of both right- and left-handers. They further observed that the incidence of arches only on digit 3 of right hands shows highly significant differences between left-handers and right-handers (P<0.001).

Coren (1994) ^11^ in his study on Canadians found that left-handers were more likely to have arches and radial loops, while fewer whorls than right-handers. The correlation of handedness and digital dermatoglyphics was most marked on the left hand, which showed significant differences on four digits except digit 1. On the right hand, handedness was associated with a digital dermatoglyphics patterns only on digit 4.

Cho (2010) ^12^, in their study on Koreans, found that both hands of left handers exhibited more arch and ulnar loop types than the right-handers and less whorl and radial loop types than the right-handers. The digital dermatoglyphic pattern of digit 3, digit 4 and digit 5 of the left hand showed a statistically significant relationship between left- and right-handed people.

In Karev's study on Bulgarian individuals ^13^, he found that whorls were significantly less frequent, and ulnar loops significantly more frequent in all digits for right-handed people when compared to left-handed people. The ulnar fluctuating asymmetries of digits 1 and 4 showed a highly significant relationship with handedness.

Rife (1955) ^16^, in his study on students at Ohio State University, USA, observed that arches were more common on the left middle finger of right-handed students than left-handed students. Left-handedness has a frequency of about 10% in the general population with a slightly higher frequency in the male population compared to the female population ^17, 18^. In our study we analyzed dermatoglyphics pattern of 70 left hander’s (35 males, 35 females) and compared it with right hander’s (35 males, 35 females). Gender wise differences in digital dermatoglyphics patterns have been established for now and then ^19^. We compared handedness wise difference of dermatoglyphics pattern in right and left hand of both male and female Sinhalese separately.

The major limitation of our study is the small sample size. Despite the small sample size, it exhibited a significant handedness wise difference of dermatoglyphics among Sinhalese. Additional research involve large sample are needed to further confirm current findings.

## Conclusion

The present study supports the hypothesis that handedness and digital dermatoglyphics are correlated in members of the Sinhalese population. Our results show that there is a statistically significant difference in fingerprint patterns between right- and left-handed people for digit 5 of the right hand and for digits 1 and 2 of the left hand in females, and digit 3 and digit 4 of the right hand and digit 4 of the left hand in males. The results of this study support the relationship between handedness and digital dermatoglyphics in the Sinhalese population. The results can be used as supporting evidence for personal identification.
