# Supplementary material for: Association between digital dermatoglyphics and handedness among Sinhalese in Sri Lanka
Source: F1000Res. 2013 Nov 4;2:111. Originally published 2013 Apr 18. [Version 3] doi: 10.12688/f1000research.2-111.v3 (PMC3917648; doi:10.12688/f1000research.2-111.v3)
Supplement: Peer review history

# Peer review: Referee response for version 3

**DOI:** 10.5256/f1000research.2852.r3981

**Published:** 2014-03-05

**Authors:** Esperanza Gutierrez Redomero

**Affiliations:** 1 Department of Zoology and Physical Anthropology, Faculty of Biology, University of Alcalá, Alcalá de Henares, Madrid, Spain

I have read this submission. I believe that I have an appropriate level of expertise to confirm that it is of an acceptable scientific standard.

# Peer review: Referee response for version 2

**DOI:** 10.5256/f1000research.1865.r2054

**Published:** 2013-10-09

**Authors:** Esperanza Gutierrez Redomero

**Affiliations:** 1 Department of Zoology and Physical Anthropology, Faculty of Biology, University of Alcalá, Alcalá de Henares, Madrid, Spain

In the Methods section, the authors say: “We recruited 50 predominant right-handers and 50 predominant left-handers after evaluating handedness” but in the Results section they say “In this study we observed the handedness-wise digital dermatoglyphics pattern distribution of 140 individuals (70 lefthanded [35 females, 35 males] and 70 right-handed [35 males, 35 females])". The error should be corrected

I have read this submission. I believe that I have an appropriate level of expertise to confirm that it is of an acceptable scientific standard.

# Peer review: Referee response for version 2

**DOI:** 10.5256/f1000research.1865.r1044

**Published:** 2013-07-08

**Authors:** Matea Zajc Petranović

**Affiliations:** 1 Institute of Anthropology, Zagreb, Croatia

The article is clearly laid out and all the key elements are present. It was significantly improved after including previous reviewers' comments.

I have read this submission. I believe that I have an appropriate level of expertise to confirm that it is of an acceptable scientific standard.

# Response

**Published:** 2013-08-01

**Authors:** Buddhika Wijerathne

**Affiliations:** Faculty of Medicine and Allied Scinces,Rajarata University of Sri Lanka,Sri Lanka, Sri Lanka

We would like to thank Matea Zajc Petranović for spending her valuable time to review the manuscript.

# Peer review: Referee response for version 1

**DOI:** 10.5256/f1000research.1272.r931

**Published:** 2013-05-07

**Authors:** Esperanza Gutierrez Redomero

**Affiliations:** 1 Department of Zoology and Physical Anthropology, Faculty of Biology, University of Alcalá, Alcalá de Henares, Madrid, Spain

The study was based on data from 100 individuals (50 left-handed [31 females, 19 males] and 50 right-handed [27 males, 23 females]). The greatest limitation of this study is the small sample size, as authors indicate; this size would be even smaller if the sample was analyzed by sex, as should have been done. The distribution of digital patterns presents sex differences in most of the samples analyzed, and so, the results cannot be accepted because they are affected by the effect of sex differences.

I have read this submission. I believe that I have an appropriate level of expertise to confirm that it is of an acceptable scientific standard, however I have significant reservations, as outlined above.

# Response

**Published:** 2013-07-08

**Authors:** Buddhika Wijerathne

**Affiliations:** Faculty of Medicine and Allied Scinces,Rajarata University of Sri Lanka,Sri Lanka, Sri Lanka

We would like to thank Professor Esperanza Gutierrez Redomero for the valuable time spent reviewing our manuscript and the important comments she has made. We have attempted to address and incorporate most of the concerns that were raised in version 2 of our article.
